# Fracture Resistance of 3D-Printed Hybrid Abutment Crowns Made from a Tooth-Colored Ceramic Filled Hybrid Composite: A Pilot Study

**DOI:** 10.3390/jfb16100375

**Published:** 2025-10-08

**Authors:** Josef Schweiger, Kurt-Jürgen Erdelt, Isabel Lente, Daniel Edelhoff, Tobias Graf, Oliver Schubert

**Affiliations:** Department of Prosthetic Dentistry, University Hospital, LMU Munich, 80336 Munich, Germany; kurt.erdelt@med.uni-muenchen.de (K.-J.E.); isabel.lente@med.uni-muenchen.de (I.L.); daniel.edelhoff@med.uni-muenchen.de (D.E.); t.graf@med.uni-frankfurt.de (T.G.); oliver.schubert@med.uni-muenchen.de (O.S.)

**Keywords:** additive manufacturing, CAD/CAM, digital dentistry, hybrid abutment crown, implant dentistry, monolithic restoration, single implant crown, 3D-printing

## Abstract

The aim of this pilot in vitro study is to investigate the fracture strength of hybrid abutment crowns (HACs) made of a 3D-printable, tooth-colored, ceramic-reinforced composite (CRC). Based on an upper first premolar, a crown was designed, and specimens were additively fabricated from a composite material (VarseoSmile Crown plus) (*N* = 32). The crowns were bonded to standard abutments using a universal resin cement. Half (*n* = 16) of the samples were subjected to artificial aging, during which three samples suffered minor damage. All specimens were mechanically loaded at an angle of 30° to the implant axis. In addition, an FEM simulation was computed. Statistical analysis was performed at a significance level of *p* < 0.05. The mean fracture load without aging was 389.04 N (SD: 101.60 N). Two HACs suffered screw fracture, while the crowns itself failed in all other specimens. In the aged specimens, the mean fracture load was 391.19 N (SD: 143.30 N). The failure mode was predominantly catastrophic crown fracture. FEM analysis showed a maximum compressive stress of 39.79 MPa, a maximum tensile stress of 173.37 MPa and a shear stress of 60.29 MPa when loaded with 389 N. Within the limitations of this pilot study, the tested 3D-printed hybrid abutment crowns demonstrated fracture resistance above a clinically acceptable threshold, suggesting promising potential for clinical application. However, further investigations with larger sample sizes, control groups, and clinical follow-up are required.

## 1. Introduction

Given their well-documented and remarkable success and survival rates, dental implants and implant-supported restorations have become indispensable elements of contemporary restorative dentistry [1,2,3]. Metal-ceramic crowns and veneered crowns with ceramic frameworks show an increased incidence of veneer fracture, i.e., “chipping” [4]. Single-implant crowns have been found to be prone to certain technical complications, inter alia the above-mentioned [1]. Due to the inherent disadvantages of veneered restorations, but also for economic considerations, monolithic materials in combination with digital workflows have increasingly come into clinical and scientific focus [5,6].

The CAD/CAM-based fabrication of dental restorations in highly standardized processes provides a range of advantages in terms of predictability, quality, and long-term prognosis, as well as efficiency [7]. Specifically, the fabrication of implant-supported restorations has profited from modern digital production processes, including data acquisition, data processing, and the fabrication of the workpiece, for instance, the single implant crown [7].

Ceramic-based hybrid abutment crowns, i.e., “HACs”, which consist of a metal base abutment and ceramic restorative materials, have proven successful in implant-supported single-tooth restorations, and have become widely used and routinely employed in implant prosthetics [8,9,10]. In this context, the fabrication of restorations made of monolithic lithium disilicate and zirconia ceramics has become established, as these materials comply with many of the requirements of modern dentistry [11,12].

Recently, additive manufacturing technologies have attracted enormous attention in dentistry and dental technology. Modern 3D-printing could help to reduce the expenditure of time and resources in the fabrication of dental restorations and is probably the manufacturing method of the future [13]. For a long time, additive manufacturing was essentially limited to the production of dental models, surgical splints, and impression trays. At present, new fields of indication are emerging, e.g., in complete prosthodontics or metal frameworks for removable partial dentures.

But as the streamlining of the workflow and economic aspects become ever more important, it seems reasonable to develop and establish additive manufacturing in the production of fixed prostheses. Although development in the field of 3D-printing of dental ceramics is progressing [14,15], additive manufacturing of zirconia-based restorations is feasible but not yet economically viable, and the production of lithium disilicate ceramics is not yet readily available for clinical applications.

This is where resin-based materials or tooth-colored hybrid materials come into consideration as a restorative option. In implant prosthodontics, the choice of restorative material directly influences long-term success. While monolithic ceramics such as zirconia and lithium disilicate have become standard due to their strength and esthetics, their rigidity may also transmit high stresses to the implant–abutment interface [16]. Materials with a lower modulus of elasticity may therefore offer stress-relieving effects on the otherwise rigid bone-implant-crown complex by implementing a kind of “damping effect” [17,18]. Their mechanical reliability, however, remains less documented, particularly when applied in combination with additive manufacturing technologies.

Against this background, 3D-printed ceramic-reinforced composites (CRCs) represent an attractive but still experimental option. They promise efficient, cost-effective fabrication of implant restorations, especially in situations with limited resources or increased esthetic demands. At present, however, clinical data are scarce, and most available evidence is restricted to short-term or provisional indications, predominantly in tooth supported restorations [19]. Recent studies have compared 3D-printed composites with milled materials in terms of accuracy, bonding, microstructure, and mechanical behavior. In general, 3D-printed crowns showed higher accuracy and fewer marginal discrepancies than milled crowns [20]. Novel resins fabricated by digital press stereolithography (DPS) demonstrated fracture resistance comparable to milled composites but lower than lithium disilicate ceramics [21]. Manufacturing technique and aging significantly affect fracture resistance, with subtractive methods yielding the highest values [22]. Printable composites display layered structures and more heterogeneous filler distribution than CAD/CAM blocks but still outperform direct composites [23]. Pull-off forces of 3D-printed crowns were sufficient for clinical use, although not significantly enhanced by airborne-particle abrasion [24]. Other comparative analyses confirmed that printed composites show lower flexural strength, hardness, and filler content compared to CAD/CAM blocks, yet remain within requirements for single-unit restorations, particularly in low-stress areas [25,26].

For emerging dental materials and technologies, pilot studies are an essential step to explore feasibility, identify critical design parameters, and generate baseline data before large-scale investigations. In the case of 3D-printed hybrid abutment crowns (HACs), no systematic evidence is currently available regarding their fracture resistance under clinically relevant loading conditions. Therefore, this pilot in vitro study was designed to provide preliminary insights into the mechanical stability of 3D-printed HACs fabricated from a tooth-colored, ceramic-filled hybrid composite, and to evaluate whether their fracture loads exceed an established acceptance threshold.

Data on the performance of these 3D-printed materials are available, yet currently scarce in scientific literature, especially in implant-supported restorations [27,28,29,30]. Therefore, the purpose of this in vitro study was to assess the fracture resistance of 3D-printed single implant crowns made from a tooth-colored ceramic filled hybrid composite before and after artificial aging. The hypotheses were that the aging process would not affect the results and that the fracture values would be within a clinically acceptable range.

## 2. Materials and Methods

**Figure 1:**
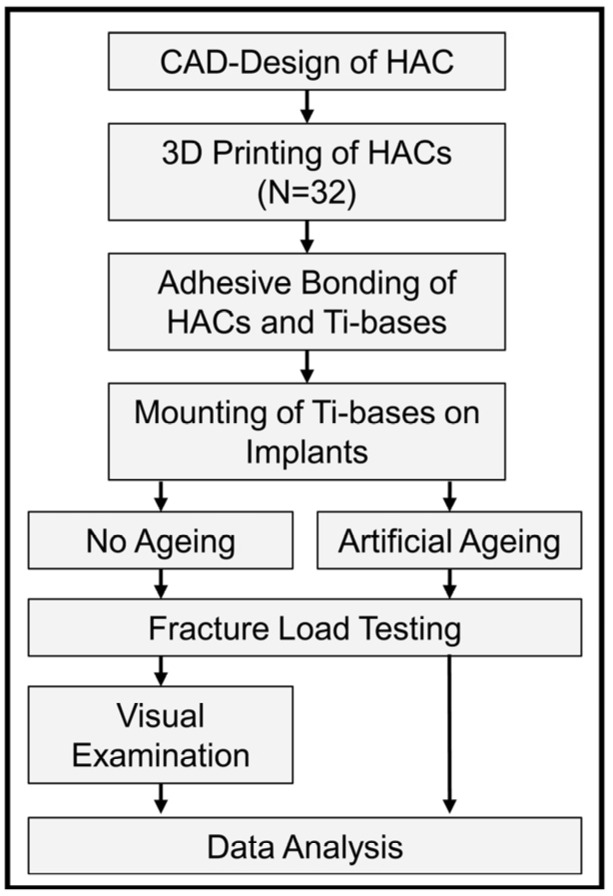
Experimental setup.

**Figure 2:**
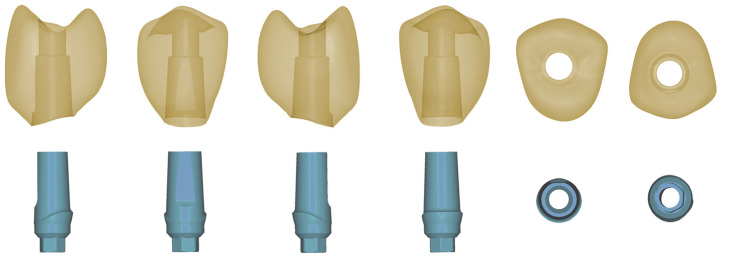
STL design data of the crown and titanium-base abutments used in this investigation from all spatial directions.

The experimental setup is given in Figure 1. The hybrid abutment crowns for this investigation were shaped to replace a maxillary premolar (DentalCAD 3.0 Galway, bego-basic-release 3.0-2021-03-25-21-05; ExoCAD, Darmstadt, Germany) and designed to fit a standard abutment as recommended by the manufacturer for this indication (Sub-Tec PLUS titanium abutment, REF 57116, implant diameter 3.25–3.75 mm; BEGO Implants; BEGO Medical, Bremen, Germany) (Figure 2). Nesting was conducted applying Asiga Composer Software (V1.2.12; MAN).

The crowns were fabricated according to the VarseoSmile Crown plus operating instructions (Asiga MAX UV 385 3D-printer; Asiga, Alexandria, Australia), post-exposed, and post-processed. The crowns were printed in shade A2 with a layer thickness of 50 μm and positioned at a 45° build orientation with support structures according to the manufacturer’s recommendations. After removal of supports and cleaning in isopropanol, post-curing was carried out in a xenon light-curing device (Otoflash, 2 × 1500 flashes under N2, NK Optik, Baierbrunn, Germany). According to manufacturer data, VarseoSmile Crown plus contains approx. 20–25 vol% ceramic fillers within a resin matrix [23]. The crowns were cleaned in an ultrasonic distilled water bath after final polishing.

To ensure a sufficient adhesive connection between crowns and abutments, the crowns were sandblasted at 1 bar and the abutments at 2 bars using 50 µm aluminiumoxide powder. Both were cleaned in an ultrasonic distilled water bath for 2 min. Clearfil Ceramic Primer Plus (Kuraray Noritake, Tokyo, Japan) was applied to both components and dried with oil-free air. Bonding was performed with Panavia V5 resin cement (Kuraray Noritake, Paste Opaque). Light-curing was carried out using a Bluephase Style LED curing unit (Ivoclar Vivadent, Schaan, Liechtenstein) with an intensity of approximately 1100 mW/cm^2^. Excess cement was removed, and margins were cured for 10 s per side.

Implants (Semados, RS 3.75 L13; BEGO Implant Systems) were mount into a special alumina fixture according to DIN EN ISO 14801:2017-03 [31] and the hybrid abutment crowns were screw-retained with 30 Ncm. The screw channel access was roughed with a diamond bur, cleaned with ethanol, and dried with oil-free air. A foam pellet was inserted, leaving 3 mm free space to the occlusal surface. Clearfil Ceramic Primer Plus (Kuraray Noritake) was applied and Clearfil Mayesty Flow A2 composite (Kuraray Noritake) was injected into the screw channel in two steps and light cured for 20 s each.

As this investigation was designed as a pilot study, the sample size was determined in a feasibility-oriented manner. A preliminary power analysis was performed on baseline fracture load data from 16 specimens. Based on the observed distribution of fracture loads and the predefined acceptance criterion, a sample size of *n* = 13 would have been sufficient to achieve a power of 0.95 at α = 0.05 (G*Power Version 3.1.9.2; Heinrich-Heine-Universität Düsseldorf, Düsseldorf, Germany) [32]. Nevertheless, 16 specimens per group were included to provide additional robustness, while acknowledging that the study was not intended to deliver definitive statistical conclusions but rather baseline data for future trials.

In total, 32 test specimens were prepared in the above-mentioned way for the present in vitro study (*N* = 32). One half (*n* = 16) was subject to artificial aging (1.2 × 10^6^ chewing cycles at 50 N, 6000 rounds of thermocycling (5° and 55 °C; dwelling time: 30 s each)) in a chewing simulator (CS-4 chewing simulator; SD Mechatronik, Feldkirchen-Westerham, Germany) [33]. The load was applied on the palatal aspect of the buccal cusp at an angle of 30° [34] to the implant axis with a 4 mm diameter stainless steel piston.

All specimens were loaded to failure with a universal testing machine (Zwick UPM 1445; Zwick, Ulm, Germany) using the software testXpert, 7.11/d (Zwick/Roell). The force was applied to palatal aspect of the buccal cusp using a steel piston (diameter: 4.0 mm) at an angle of 30°. Crosshead-speed was 0.5 mm/min at a pre-load of 0 N. The defined end point was a sudden drop of force more than 30% or a deflection of ≥2.0 mm. The non-aged specimens were unmounted and examined for deformation around the implant-abutment-interface (IAI) using an optical microscope (OM) (Axioskop 2MAT, Carl Zeiss, Jena, Germany) at 10× magnification.

All experiments were performed in the dental research laboratory of the Department of Prosthetic Dentistry, University Hospital, LMU Munich. All testing devices were calibrated according to the manufacturers’ specifications prior to use. Masking of the samples was not performed, as all crowns were produced using standardized CAD/CAM workflows under identical conditions.

A value of 290.1 N was calculated as an acceptance criterion. This was calculated from the mean fracture load of 487.3 N for the Implant S with a diameter of 3.75 mm (BEGO Implant Systems) in accordance with DIN EN ISO 14801:2017-03—as specified by the manufacturer—and the ratio of the lever arm in the present study (9.23 mm) and the lever arm in the ISO standard (5.5 mm): 487.3 N/(9.23 mm/5.5 mm) = 290.1 N.

In addition to the in vitro testing, a FEM simulation was performed to provide preliminary insights into stress distribution within the crown-abutment complex. SolidWorks 3D CAD (Dassault Systèmes, Vélizy-Villacoublay, France) was used for reverse engineering of the STL data, and Ansys 19.0 R2 software (Ansys, Canonsburg, PA, USA) was employed for the calculations. The elastic moduli adopted were 4090 MPa for the composite crown and 105 GPa for the titanium abutment and implant. The cement layer was not modeled separately, since the cement resins elastic modulus resembles the one of the crown material. A 4 mm steel sphere (220 GPa) simulated the piston contact, and the mean fracture load of 389 N was applied. The objective was not to achieve full predictive accuracy, but rather to visualize principal stress patterns under loading in this pilot study.

The obtained data of the fracture values of the hybrid abutment crowns were imported into the statistical program SPSS (Statistics 25.0, SPSS Inc., Stanford, CA, USA) for statistical processing, prepared for analysis and evaluated afterwards. The Kolmogorov–Smirnov test was used to check the values for normal distribution. Mean/median values and the standard deviations/IQRs were determined. To test for significance between the two groups, the Mann–Whitney U test was used. To compare each group to the acceptance criterion, Wilcoxon test was applied. The significance level was set at 5% (*p* < 0.05).

## 3. Results

**Figure 3:**
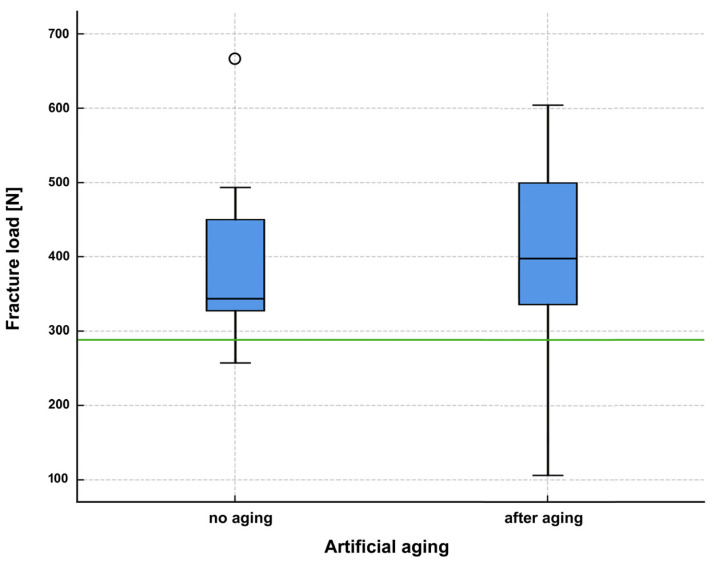
Boxplot diagram of fracture load values testing in the non-aged and aged groups. The green line depicts the acceptance load at 290.1 N.

**Figure 4:**
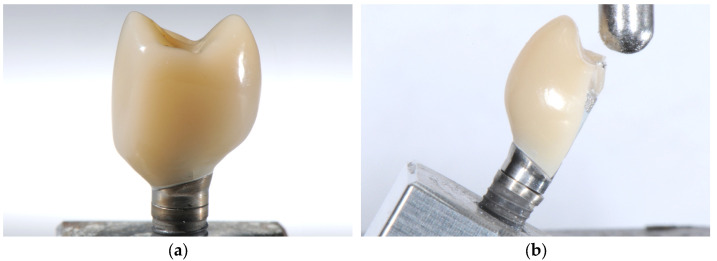
Results of the fracture load tests: (**a**) HAC after artificial aging showing a crack at the transition between crown material and titanium base abutment (random sample). (**b**) HAC fractured after load to failure testing (random sample).

Fracture values are given in Figure 3 and Table 1 and Table 2. All specimens of the aged group survived cyclic loading and thermocycling. Three specimens were found to be fractured within the restorative material. Two crowns showed damage in the cusp area, which resulted in a significant reduction in the fracture load. One showed a fracture at the junction between the composite material and the titanium base abutment (Figure 4a), which did not significantly affect the mechanical stability.

In the non-aged group (Table 1), 14 crowns displayed a catastrophic fracture after loading, while in two specimens the retention screws fractured. Mean failure load was 389.04 N (SD: 101.6 N) with a maximum at 664.13 N and a minimum at 257.2 N. Median was 343.95 N with an interquartile range (IQR) 136.45 N. The typical failure pattern of the fractured crowns, that comprised a vertical fractured along the buccal aspect with exposition of the titanium base abutment is displayed in Figure 4b.

After artificial aging, three specimens displayed visible cracks before load-to-failure testing: one mesio-occlusal crack at the buccal cusp (specimen 31), one distal marginal crack of 0.6 mm length (specimen 4), and one mesio-occlusal crack extending from the cusp (specimen 8). Two of these led to reduced fracture loads, while one had no measurable effect.

In the aged group (Table 2), specimens failed at an average of 391.19 N (SD: 143.3 N) with a maximum at 603.89 N and a minimum at 106.48 N. Median was 397.97 N with an interquartile range (IQR) 177.72 N. While the screws fractured in three specimens and a cohesive failure pattern was observed in one crown, the remaining twelve crowns fractured like the crowns in the non-aged group.

Since normal distribution could only be assumed for the aged group, non-parametric tests were employed for statistical analysis of the fracture loads. The Mann–Whitney U test for independent samples yielded a value of 0.423, i.e., there was no significant difference between the non-aged and the aged group.

Statistical comparison also revealed significant differences between the acceptance criterion and the non-aged specimens (*p* < 0.001) and the aged group (*p* = 0.026). The results in both groups were considerably higher than the level specified as an acceptance criterion.

**Figure 5:**
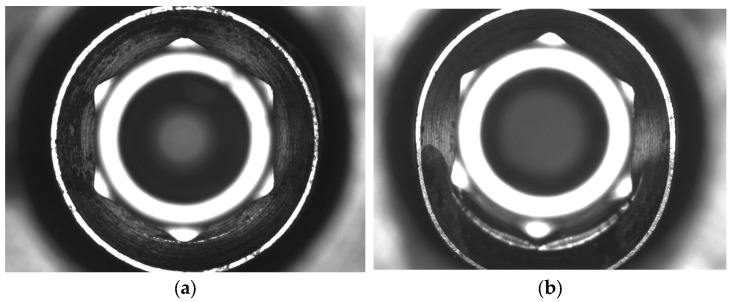
Optical microscope images (10× magnification) of the deformation at the IAI and comparison of: (**a**) a sample with a fracture load value of 257.2 N and (**b**) a sample with a value of 472.99 N.

**Figure 6:**
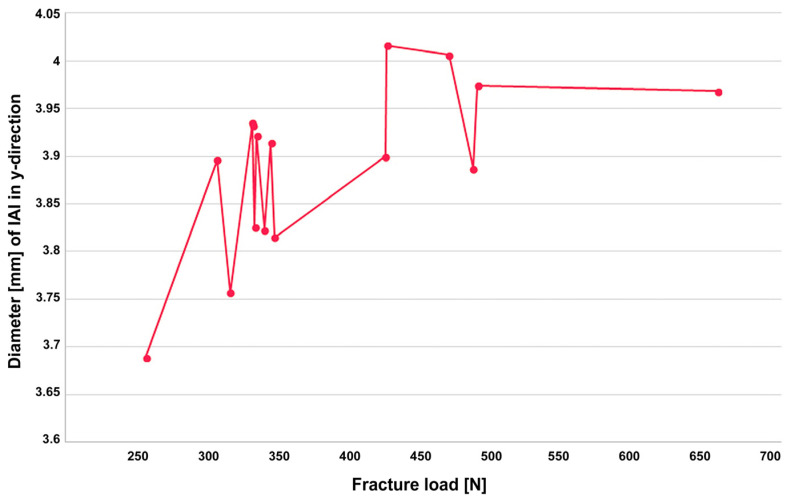
Diameter [mm] of the implant abutment connections (IAI) in y-direction as after fracture load testing [N].

The assessment of non-aged specimens for deformation around the IAI (Figure 5a,b) using an optical microscope demonstrated an evident connection and positive correlation with the measured fracture load (Figure 6). The largest deformation observed was 326 µm in y-direction.

**Figure 7:**
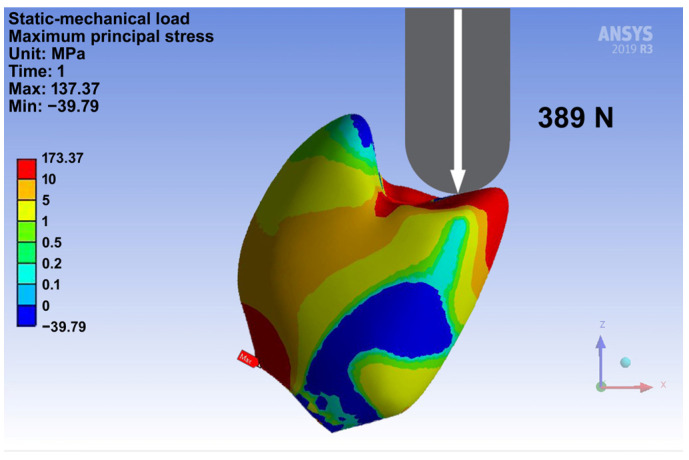
FEM simulation of load testing.

The exploratory FEM analysis indicated a maximum compressive stress of 39.79 MPa and a maximum tensile stress of 173.37 MPa under a load of 389 N. Shear stress reached a maximum of 60.29 MPa (Figure 7).

**Figure 8:**
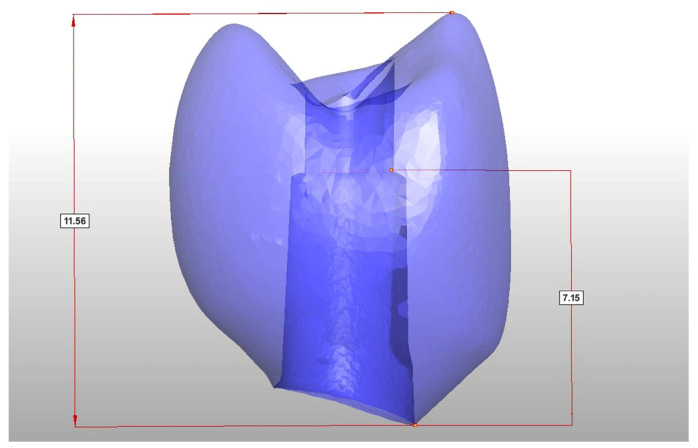
The dimensions of the crown, representing a crown-to-abutment height of 1.6.

Based on the data of this study, it can be concluded that the ratio of crown height to abutment height has a significant influence on the fracture load of hybrid abutment crowns. The ratio of crown height to the height of the bonding surface of the abutment should not exceed a value of 1.6 (Figure 8).

## 4. Discussion

Modern implant-supported dental restorations benefit greatly from digital workflows and advanced materials, providing enhanced efficiency and predictability. Hybrid abutment crowns (HACs), which combine a titanium base abutment with a tooth-colored restorative component, have proven to be an effective and reliable option for implant-supported single crowns. Ceramic materials, such as lithium disilicate and zirconia, offer excellent mechanical and esthetic properties as well as high biocompatibility [35,36,37]. The use of titanium as a CAD/CAM abutment material has become well established, and the adhesive connection between ceramic restorative materials and titanium abutments has proven highly reliable [38].

A recent investigation assessed the clinical performance of monolithic HACs by means of the “Functional Implant Prosthodontic Score” (FIPS [39]). The HACs made from lithium disilicate presented a high survival rate of 97.5% over more than 3.5 years and an overall FIPS score of 8.68 out of 10 (SD: 1.12), with only minor complications [10]. Other research confirms that monolithic trans-occlusal screw-retained HACs made from tooth-colored materials, especially lithium disilicate [40,41,42], have become a dependable solution in implant prosthodontics.

Monolithic zirconia is also a feasible option for the manufacture of HACs, given that modern zirconia materials offer improved optical properties [43], superior biocompatibility [36], and likewise very efficient workability. However, concerns remain that their high rigidity may transfer excessive stresses to the titanium base or even the implant itself, potentially leading to mechanical failures [16,28]. A scientific evaluation of this issue under clinical conditions is needed.

Resin based materials with advanced characteristics and features, such as resin matrix ceramics (RMC), might make a useful contribution to single-implant restorations, [8,17,18] particularly due to their lower elastic modulus, which could allow stress relief within the implant-crown complex. Nevertheless, preliminary in vivo studies indicate higher failure rates compared to ceramics, and further clinical data are needed to validate their long-term performance [3].

In this context, additive manufacturing (3D-printing) has gained increasing interest due to its potential for streamlined, cost-efficient production and precise replication of complex geometries. The capability to directly fabricate crowns with internal connections for titanium bases makes this technique particularly appealing for implant-supported restorations.

In the present study, all crowns exceeded the acceptance threshold of 290.1 N, even after artificial aging. The predefined acceptance threshold of 290.1 N was derived from manufacturer data and lever-arm relations of the tested implant system. While this pragmatic approximation provides a useful reference point, its clinical relevance is limited. The forces fall within clinically relevant ranges [44], which, however, may be exceeded in cases of parafunctional habits. This emphasizes the need for careful patient selection and preventive measures, such as occlusal splints. The consistently higher fracture loads measured in the present study, exceeding the threshold, indicate that the tested crowns are likely to withstand physiologic and even elevated functional loads.

The results are in contrast to the findings of Graf et al., who reported that seven out of ten HACs made from the same material did not survive artificial aging [28]. This difference might be attributed to a merely unfavorable crown-to-abutment height ratio, which did not correspond to the material’s specific requirements, among other differences in the experimental setup. Comparing 3D-printed and conventional PMMA provisional single crowns de Souza et al. showed that they performed comparably on anterior implants, with the exception of more catastrophic fractures in the 3D-printed group. Despite shorter operating times for 3D printed crowns, patient satisfaction did not differ, suggesting that both approaches are clinically acceptable for provisional use [27]. Othmann et al. concluded from their in vitro investigation that milled molar crowns showed significantly higher fracture strength than 3D-printed ones, while no significant difference was found for incisors. Clinically, both milled and 3D-printed crowns withstood masticatory forces, supporting the use of 3D-printed crowns as a viable option for long-term provisional restorations [29]. Donmez et al. even found that implant-supported 3D-printed composite crowns showed better marginal fit than milled ones and similar fracture forces [30].

Notably, some aged specimens in the present study exhibited minor defects (such as small cracks) after cyclic loading. These were not advantageous but had little effect on fracture strength, and the crowns still demonstrated fracture loads significantly above the limit value, underscoring the robustness of the material and design. Furthermore, the optical microscopy analysis revealed that higher fracture loads were directly associated with increasing deformation at the IAI, indicating a transition from elastic to plastic behavior at loads above 300 which is consistent with the implant manufacturer’s recommendation. However, the clinical relevance of these implant-abutment interface deformations observed at higher fracture loads remains to be elucidated. Therefore, it should be further investigated in future in studies, e.g., dynamic masticatory simulations and prospective clinical trials with long-term follow-up.

Another critical factor identified in this study is the ratio between crown height and abutment height. Maintaining this ratio below 1.6 proved essential for achieving sufficient fracture resistance and minimizing bending moments. This design parameter should be carefully considered in clinical workflows to optimize mechanical stability, especially when using resin-based or hybrid materials.

The study was also intentionally designed as a worst-case scenario, including an off axis loading angle of 30°, a small implant diameter, and abutments lacking a supporting shoulder. These challenging conditions were chosen to rigorously evaluate the crowns’ performance under extreme mechanical stress. The fact that the crowns withstood these conditions reinforces their potential clinical applicability and reliability.

Beyond implant-specific investigations, the present study adds to the growing body of evidence on 3D-printed composites. Several recent studies have systematically compared milled and 3D-printed composites in terms of accuracy, bonding, microstructure, and fracture resistance. Kakinuma et al. demonstrated that 3D-printed crowns exhibited higher trueness and fewer marginal discrepancies than milled restorations, whereas milling was associated with dimensional deviations, particularly at cusps, and with internal grooves from offset correction [20]. Abad-Coronel et al. reported that lithium disilicate restorations achieved the highest fracture resistance, hybrid ceramics the lowest, and polymer-based materials (milled Cerasmart and a 3D-printed resin using digital press stereolithography) intermediate and comparable values, suggesting that additive techniques can approach the performance of subtractive ones, though ceramics remain superior [21]. In a broader comparison, Güney et al. confirmed that subtractive manufacturing generally yielded the highest fracture resistance for both crowns and fixed partial dentures, but also observed that thermal aging reduced the strength of subtractive restorations and diminished differences between manufacturing methods [22]. On a microstructural level, Prause et al. showed that CAD/CAM blocks exhibit highly homogeneous filler distribution, direct composites significant inhomogeneities, and printable composites a layered intermediate pattern [23]. Graf et al. (2022) further demonstrated clinically sufficient pull-off retention forces for 3D-printed VarseoSmile Crown plus crowns, with no significant additional benefit from airborne-particle abrasion [24]. Grzebieluch et al. found that 3D-printed composites generally had lower flexural strength, hardness, and filler content compared with CAD/CAM blocks, and that build orientation strongly influenced strength [25]. Similarly, Sahin et al. concluded that subtractive composites outperformed printed ones in flexural strength and hardness, but noted that printed resins still fulfilled DIN EN ISO 6872:2015 [45] requirements for single-unit anterior restorations, while appearing less suitable for posterior high-load applications [26]. Collectively, these findings support the present observation that 3D-printed composites may be considered clinically feasible for selected indications but cannot yet rival ceramics or CAD/CAM blocks in long-term reliability, particularly for full-coverage posterior restorations.

This investigation has limitations that reflect its pilot character. No CAD/CAM composite control group was included, and fractographic inspection in this study was limited to optical microscopy; no stereomicroscopy or SEM was performed. Moreover, the results of 3D printing always reflect and are influenced by both the material and the printing technique. However, general implications for mechanical stability can still be deduced, providing valuable guidance for future development and material optimization. In the FEM, the cement layer was omitted, since the resin cement has an elastic modulus comparable to the crown material. Moreover, the FEM approach does not fully capture the rheological (flow) behavior of polymer-based materials, including their time- and load-dependent viscoelastic and plastic deformation. The fracture patterns observed during mechanical testing differed from those predicted by FEM. While FEM suggested crack initiation at maximum tensile stresses, the crowns predominantly failed along an oblique line from the buccal cusp towards the cervical area, indicating shear-driven fracture and plastic deformation consistent with the ductile behavior of the hybrid composite material. Nevertheless, FEM provided useful preliminary visualization of stress concentrations that qualitatively reflected the observed fracture lines. These discrepancies highlight the limits of linear-elastic FEM and underscore the need for more advanced modeling. Within this pilot investigation, FEM therefore served only as an exploratory tool to support, but not to replace, the experimental findings.

In addition, the sample size was limited to a feasibility level. Taken together, these factors emphasize that the present work provides preliminary rather than definitive evidence. Future studies should address these limitations.

It should be noted that the bonding protocol used in this study reflected the manufacturer’s official recommendation for VarseoSmile Crown plus at the time of the investigation. The protocol has since been revised, now recommending adherence to the instructions of the resin cement manufacturer to optimize chemical polymerization. This change underscores the dynamic nature of bonding strategies for emerging printable restorative materials.

Despite these limitations, the present investigation provides valuable baseline data on the mechanical behavior of 3D-printed hybrid abutment crowns and helps to define critical design parameters for future studies and clinical applications. Overall, the findings indicate that 3D-printed hybrid abutment crowns, when carefully designed and properly bonded, may represent a promising option for implant-supported single restorations. However, conclusions regarding clinical use must be drawn with caution. A recent review on 3D-printed ceramic-reinforced composites concluded that, based on current evidence, machinable materials are most suitable for situations involving lower occlusal forces, while 3D-printed ceramic-reinforced composites are primarily recommended for provisional use. Recent systematic analyses further emphasize that machinable CAD/CAM CRCs may be applied in low-stress partial restorations, whereas first-generation 3D-printed CRCs are best regarded as long-term provisionals due to their higher wear and surface degradation. Both remain inferior to ceramics in terms of long-term stability, which should therefore remain the material of choice for definitive full-coverage restorations until robust long-term clinical data become available [19]. Further long-term in vivo investigations are warranted to confirm the results of the present study and to assess long-term clinical behavior under functional loading.

## 5. Conclusions

Within the limitations of this pilot in vitro study, it can be concluded that the tested 3D-printed hybrid abutment crowns made from a tooth-colored ceramic-filled hybrid composite material demonstrated sufficient fracture resistance both before and after artificial aging. The crowns consistently exceeded the defined acceptance load of 290.1 N, even under a worst-case scenario with an off axis loading angle and small implant diameter. The additional design parameter of maintaining a crown-to-abutment height ratio below 1.6 contributed to the mechanical stability and should be regarded as an important clinical guideline. Furthermore, the observed fracture patterns indicated a shear-dominated failure rather than a purely brittle fracture, highlighting the material’s capacity for energy absorption. These findings, therefore, provide preliminary evidence that 3D-printed HACs may be suitable for further development, but validation in larger, controlled in vitro and prospective in vivo studies is required before definitive clinical recommendations can be made.

## Figures and Tables

**Table 1:** Fracture load, deformation at the IAI in spatial directions x and y, and mode of failure for the non-aged specimens. Acceptance load was defined as 290.1 N.

No	Fracture Load [N]	X [mm]	Y [mm]	Failure Mode
1	257.2	3.627	3.689	catastrophic crown fracture
2	308	3.666	3.896	catastrophic crown fracture
3	317.2	3.64	3.757	catastrophic crown fracture
4	332.9	3.676	3.935	catastrophic crown fracture
5	333.3	3.666	3.932	catastrophic crown fracture
6	334.8	3.694	3.826	catastrophic crown fracture
7	336.2	3.647	3.922	catastrophic crown fracture
8	341.5	3.681	3.823	catastrophic crown fracture
9	346.5	3.664	3.914	catastrophic crown fracture
10	348.9	3.664	3.815	catastrophic crown fracture
11	427.6	3.63	3.9	catastrophic crown fracture
12	428.9	3.648	4.016	screw fracture
13	472.99	3.628	4.006	screw fracture
14	490.07	3.641	3.887	catastrophic crown fracture
15	493.5	3.687	3.974	catastrophic crown fracture
16	664.1	3.607	3.968	catastrophic crown fracture

**Table 2:** Fracture load, deformation at the IAI in spatial directions x and y, and mode of failure for the aged specimens. Acceptance load was defined as 290.1 N.

No	Fracture Load [N]	Failure Mode
1	522.14	catastrophic crown fracture
2	472.28	screw fracture
3	540.08	catastrophic crown fracture
4	176.02	catastrophic crown fracture
5	357.69	catastrophic crown fracture
6	106.48	cohesive crown fracture
7	313.15	catastrophic crown fracture
8	518.25	catastrophic crown fracture
9	603.89	screw fracture
10	493.01	catastrophic crown fracture
11	376.88	catastrophic crown fracture
12	465.01	screw fracture
13	158.19	catastrophic crown fracture
14	390.81	catastrophic crown fracture
15	378.26	catastrophic crown fracture
16	405.12	catastrophic crown fracture

## Data Availability

The original contributions presented in the study are included in the article, further inquiries can be directed to the corresponding author.
